# Efficacy and Safety of Intraosseous Versus Intravenous Antibiotic in Primary and Revision Total Joint Arthroplasty: A Systematic Review and Meta-Analysis

**DOI:** 10.3390/medicina61101750

**Published:** 2025-09-25

**Authors:** Sunwoo Lee, Jiyun Kang, Yonggyun Moon, Jaeyoung Hong, Hyoungtae Kim, Suenghwan Jo

**Affiliations:** 1 College of Medicine, Chosun University, Gwangju 61452, Republic of Korea; doer2103@gmail.com (S.L.); jiyun.kang37@gmail.com (J.K.); moonyonggyun2146@gmail.com (Y.M.); jyhveritas@gmail.com (J.H.); kht2769@naver.com (H.K.); 2 Department of Orthopedic Surgery, Chosun University Hospital, Gwangju 61453, Republic of Korea

**Keywords:** antibiotics, intraosseous drug administration, prosthesis joint infection, total knee arthroplasty, total hip arthroplasty

## Abstract

*Background and Objectives*: Periprosthetic joint infection (PJI) is one of the most serious complications following total joint arthroplasty (TJA), leading to poor functional outcomes and increased healthcare burden. Intraosseous (IO) regional antibiotic prophylaxis has emerged as a promising method for infection prevention, offering potential advantages over conventional intravenous (IV) systemic administration. This study aimed to systematically evaluate the clinical evidence on the efficacy and safety of IO prophylaxis in TJA. *Materials and Methods*: A comprehensive literature search was conducted in PubMed, Embase, Scopus, Web of Science, and the Cochrane Library up to 23 June 2025. Eligible studies included randomized controlled trials and observational studies reporting outcomes of IO antibiotic administration in TJA. Primary endpoints were systemic and local antibiotic concentrations (bone and fat tissue), the incidence of PJI, and complication profiles. Risk of bias was assessed using the ROB-2 and ROBINS-I tools, and meta-analyses were conducted using RevMan v 7.2.0. *Results*: Fifteen studies (eight RCTs, one prospective study, and six retrospective studies) were included for qualitative synthesis, of which ten were included in the meta-analysis. As compared with IV, IO administration resulted in significantly higher local antibiotic concentrations in bone (MD: 15.52 μg/g; 95% CI: 8.60–22.45; *p* < 0.0001) and fat tissue (MD: 18.15 μg/g; 95% CI: 12.86–23.45; *p* < 0.0001). IO prophylaxis was associated with a significantly lower PJI rate (OR: 0.26; 95% CI: 0.12–0.57; *p* = 0.008) without a significant difference in the incidence of complications (*p* = 0.66). *Conclusions*: IO antibiotic prophylaxis is an effective and safe strategy for infection prevention in total joint arthroplasty. By achieving superior local antibiotic concentrations and reducing PJI rates without increasing complications, this approach holds promise for broader clinical application.

## 1. Introduction

Periprosthetic joint infection (PJI) is a serious yet relatively infrequent complication of total knee arthroplasty (TKA) and total hip arthroplasty (THA), with an incidence of 1–2% following primary procedures [1]. Despite its low occurrence, PJI is a major cause of arthroplasty failure and revision, leading to substantial patient morbidity and healthcare costs [2,3]. With an aging population and increasing arthroplasty volume, the absolute number of PJIs is expected to rise, highlighting the need for improved prophylactic strategies [4,5].

Since the 1970s, intravenous (IV) antibiotic prophylaxis has constituted the standard of antibiotic prophylaxis because of its demonstrated efficacy in reducing periprosthetic joint infection (PJI) [6]. First-generation cephalosporins have been the most commonly used agents for intravenous (IV) prophylaxis; however, rising antimicrobial resistance has led to increasing consideration of vancomycin as an alternative [7,8]. Vancomycin has shown efficacy in the treatment of infections caused by MRSA and coagulase-negative staphylococci (CoNS) [9]. However it presents pharmacologic challenges: it requires slow infusion, has limited local tissue penetration, and carries a risk of nephrotoxicity and other adverse effects [10]. Intraosseous (IO) regional antibiotic administration has emerged as a promising alternative to enhance local drug delivery while minimizing systemic exposure [11]. By injecting antibiotics directly into cancellous bone, IO prophylaxis produces significantly higher local tissue concentrations and simultaneously minimizes systemic concentrations. This technique was shown to be more effective and safe in surgical settings than intravenous (IV) prophylaxis [12,13].

Recent systematic reviews have reported that IO antibiotic delivery achieves higher tissue concentrations and reduces PJI rates more effectively than standard IV prophylaxis in patients undergoing TJA [12,13,14,15]. These reviews provided successful syntheses across various TJA procedures, particularly TKA. However, apart from studies in TKA, much of the included evidence involving aseptic revision TKA or THA that was heterogeneous or derived from small-scale retrospective cohorts. Therefore, there appears to be a need for updated meta-analyses incorporating recently published studies.

The aim of this study is to systematically evaluate the effectiveness and safety of intraosseous (IO) regional antibiotic prophylaxis in various TJA procedures through the assessment of tissue concentration, PJI rate, and complication incidence. Through the meta-analysis of recent clinical studies, we seek to determine whether IO administration is a superior infection prevention strategy and whether it can be considered a reliable adjunct or alternative to conventional IV prophylaxis in routine arthroplasty practice.

## 2. Materials and Methods

This systematic review was performed in accordance with PRISMA (Preferred Reporting Items for Systematic Reviews and Meta-Analyses statement) [16,17] guidelines and NECA (National Evidence-based Healthcare Collaborating Agency)’s guidance [18]. The protocol for this review was pre-registered on PROSPERO (registration number: CRD420251065554).

### 2.1. Literature Search

A comprehensive electronic search was performed on 23 June 2025 in five bibliographic databases—PubMed, Embase, Scopus, Web of Science, and the Cochrane Library—without date restrictions. The search query combined keywords as follows: ([Arthroplasty, Replacement, Hip] OR [Arthroplasty, Replacement, Knee]) AND ([Hip Prosthesis] OR [Knee Prosthesis]) AND ([Anti-Bacterial Agents] OR [Anti-Infective Agents, Local]) AND (Infusions, Intraosseous) (Supplementary S1). The reference lists of the included studies were also reviewed to identify any additional relevant articles.

### 2.2. Eligibility Criteria

The inclusion of studies was determined based on the following PICO criteria: P (Population): adult patients undergoing primary or revision THA or TKA; I (Intervention): intraosseous administration of prophylactic antibiotics during surgery; C (Comparison): patients who received intravenous antibiotic prophylaxis; O (Outcomes): antibiotic concentration in local tissue and fat tissue, minimal inhibitory concentrations (MICs), incidence of prosthetic joint infection (PJI), and antibiotic-related complications. Randomized controlled trials (RCTs) and observational studies (prospective or retrospective) were included, whereas reviews, comments, case reports, descriptive studies, or news articles were excluded. Furthermore, non-English, non-human, and non-clinical studies were also excluded.

### 2.3. Study Selection

Prior to screening, all references identified through database searches were deduplicated using EndNote (version 21.5). Two researchers (S.L. and J.K.) independently screened the titles and abstracts of the deduplicated studies based on the eligibility criteria. Subsequently, full-text screening was conducted to determine the final set of included studies. Any discrepancies were resolved through discussion with the senior author (S.J.).

### 2.4. Data Extraction

Data extraction was performed by one author (S.L.), and the extracted data were independently reviewed and verified by the other members of the research team. The extracted demographic and clinical data were summarized as follows: demographic variables included sample size (*n*), mean age (years), sex distribution (%), average body mass index (BMI, kg/m^2^), tourniquet time (minutes), minimum follow-up duration (months), American Society of Anesthesiologists (ASA) score, and serum creatinine level (mg/dL). Outcome data for meta-analysis included tissue concentrations (bone and fat), serum concentrations, prosthetic joint infection rate, and incidence of complications.

### 2.5. Risk-of-Bias Assessment

The independent researchers (L.S.W. and J.K.) assessed the risk of bias for included studies. The risk of bias in randomized controlled trials (RCTs) was assessed using the ROB-2 tool (a revised Cochrane risk-of-bias tool for randomized trials), while non-randomized studies were evaluated with the ROBINS-I tool (risk of bias in non-randomized studies of interventions). Any disagreements in the assessment were resolved by discussion between reviewers and a third reviewer (J.H.).

### 2.6. Statistics

For statistical analysis, mean difference and standard mean difference were used for continuous variables, while odd ratio was applied to categorical variables. The statistical pooling of data was conducted using Chi-square and I^2^ analysis. Depending on the level of heterogeneity, either a fixed effects or random effects model was applied. All data management and meta-analysis tasks were performed using RevMan version 7.2.0. (Copenhagen, The Cochrane Collaboration). When mean differences (MDs) and standard deviations (SDs) required for meta-analysis were not available in the included studies, we estimated them using an online calculator based on the available data such as medians and quartile ranges [19,20,21,22].

## 3. Results

### 3.1. Study Identification

**Figure 1:**
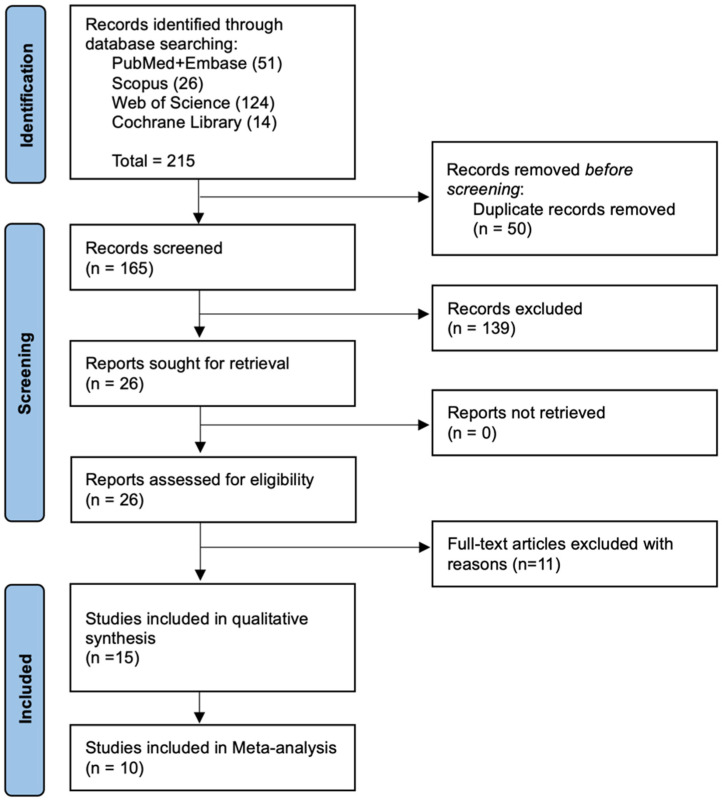
PRISMA flow diagram.

The PRISMA flowchart is depicted in Figure 1. The initial search yielded a total of 215 studies (51 from PubMed and Embase, 26 from Scopus, 124 from Web of Science, and 14 from Cochrane Library). The title and abstract screening yielded 26 potentially eligible studies, and full-text assessment resulted in 15 studies meeting the PICO inclusion criteria. Finally, 8 randomized controlled trials (one involving total hip arthroplasty) [11,23,24,25,26,27,28,29], five cohort studies (all focused on total knee arthroplasty) [30,31,32,33,34], and two consecutive series [35,36] were included. Of these, 10 studies were included in the meta-analysis.

### 3.2. Study Characteristics

Baseline demographic and clinical characteristics—including age, sex distribution, body mass index (BMI), smoking status, comorbidity prevalence (e.g., diabetes, chronic renal failure, rheumatoid arthritis, liver disease, and human immunodeficiency virus infection), and American Society of Anesthesiologists (ASA) classification—were reported across the included studies (Table 1). These characteristics were generally comparable between the intraosseous (IO) and intravenous (IV) groups, although the completeness of reporting varied. Where available, laboratory parameters such as serum creatinine, serum albumin, and glomerular filtration rate (GFR) were also extracted.

### 3.3. Risk of Bias

**Figure 2:**
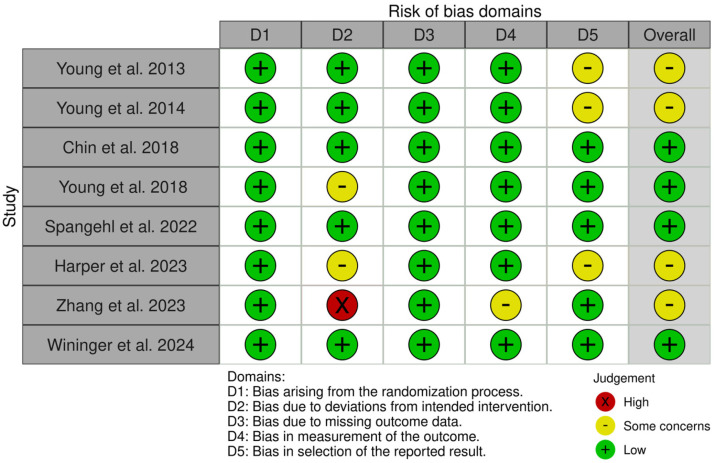
Risk of bias according to ROB-2 in RCTs [11,23,24,25,26,27,28,29].

**Figure 3:**
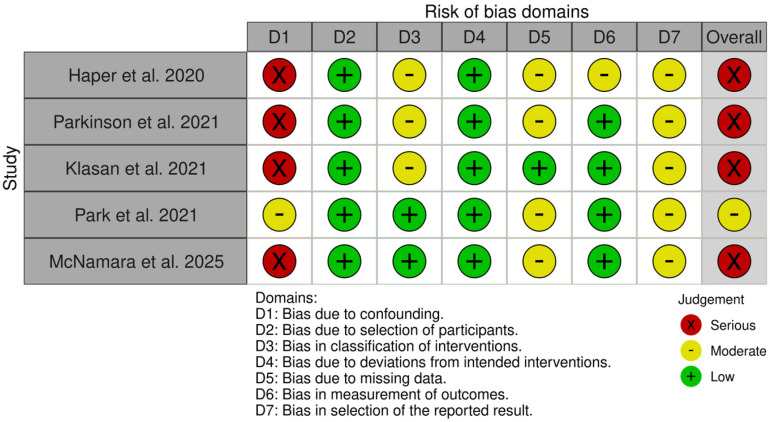
Risk of bias according to ROBINS-I V2 tool in observational studies [30,31,32,33,34].

The results of the risk-of-bias assessment are depicted in Figure 2 and Figure 3. Risk of bias was evaluated for eight RCTs [11,23,24,25,26,27,28,29] and five cohort studies [30,31,32,33,34]; case series without a control group, such as Lachiewicz et al. [35] and Christopher et al. [36], were not assessed. Using the ROB-2 tool, four RCTs were judged to have a low risk of bias and four were considered to have ‘some concerns’. For the five cohort studies assessed with the ROBINS-I tool, one study was rated as ‘moderate’ risk, while the remaining four were judged as ‘serious’ risk.

### 3.4. Efficacy of IO Antibiotic Prophylaxis Achieving Higher Antibiotic Concentrations

#### 3.4.1. Antibiotic Concentration in Local Bone Tissues

**Figure 4:**
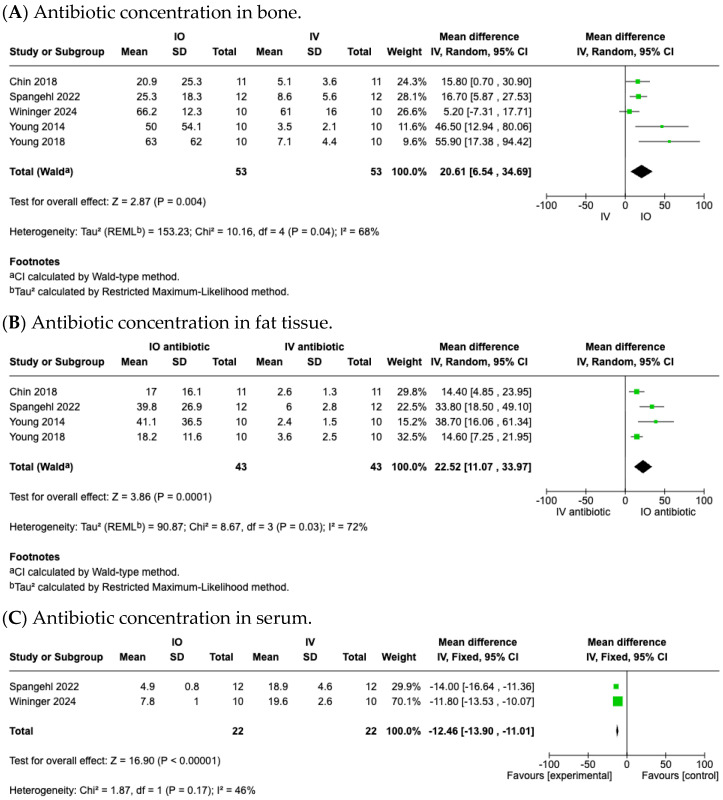
Forest plot comparing vancomycin concentration between IO and IV antibiotic prophylaxis groups: (**A**) in bone tissue; (**B**) in fat tissue; (**C**) in serum [23,24,25,26,29].

A total of seven studies [11,23,24,25,26,27,29] reported the antibiotic concentration in local bone tissues (Table 2). Five studies [23,24,25,26,29] and 106 cases were included in the meta-analysis, all of which were RCTs (Table 1 and Table 2). Pooled data from these studies showed that the antibiotic concentration in local bone tissue was significantly higher in the IO group than IV group (MD, 20.61; 95% CI, 6.54 to 34.69; *p* = 0.004; I^2^ = 68%; random effects model) (Figure 4A).

In the study by Young et al. [11], both intraosseous (IO) and intravenous (IV) groups received 1 g of cefazolin; the mean antibiotic concentration in local bone tissue was also significantly higher in the IO group (130 μg/g) compared with the IV group (11.4 μg/g; *p* < 0.01). And in Harper et al. [27], which included only patients undergoing THA, the IO group demonstrated a higher vancomycin concentration in acetabular bone reaming (*p* = 0.001) and in the intramedullary femoral bone (*p* = 0.053).

#### 3.4.2. Antibiotic Concentration in Local Fat Tissues

A total of six studies [11,23,24,25,26,28] reported the antibiotic concentration in local fat tissues (Table 2). Four studies [23,24,25,26] and 86 cases were included in the meta-analysis, all of which were RCT (Table 1 and Table 2). Pooled data from these studies showed that the antibiotic concentration in local fat tissue was significantly higher in the IO group than IV group (MD, 22.52; 95% CI, 11.07 to 33.97; *p* = 0.0001; I^2^ = 72%; random effects model) (Figure 4B).

In the study by Young et al. [11], the mean antibiotic concentration in subcutaneous fat was also significantly higher in the IO group (186 μg/g) compared with the IV group (10.6 μg/g; *p* < 0.01). In the study by Zhang et al. [28], patients undergoing TKA received 2 g of cefazolin in both the IO and IV groups. The IO group demonstrated a higher antibiotic concentration in subcutaneous fat (247.9 ± 64.9 μg/g) compared with the IV group (11.4 ± 1.9 μg/g).

#### 3.4.3. Antibiotic Concentration in Serum

A total of five studies [23,24,26,27,29] reported antibiotic concentration in serum (Table 2). Two studies [26,29] and 44 cases were included in the meta-analysis, all of which were RCT (Table 1 and Table 2). Pooled data from these studies showed that the serum antibiotic concentration was significantly lower in the IO group than IV group (MD, −12.46; 95% CI, −13.90 to −11.01; *p* < 0.00001; I^2^ = 46%; fixed effects model) (Figure 4C).

In Chin et al. [11] and Young et al. [23], the mean serum antibiotic concentration is also lower in the IO group compared with the IV group (*p* < 0.01). In the study by Harper et al. [27], which investigated patients undergoing THA, a significant reduction was observed both at the start of the procedure and at the time of closure (*p* < 0.001).

### 3.5. Safety of IO Antibiotic Prophylaxis in Preventing PJI and Complications

#### 3.5.1. Prosthesis Joint Infection (PJI) Rate

**Figure 5:**
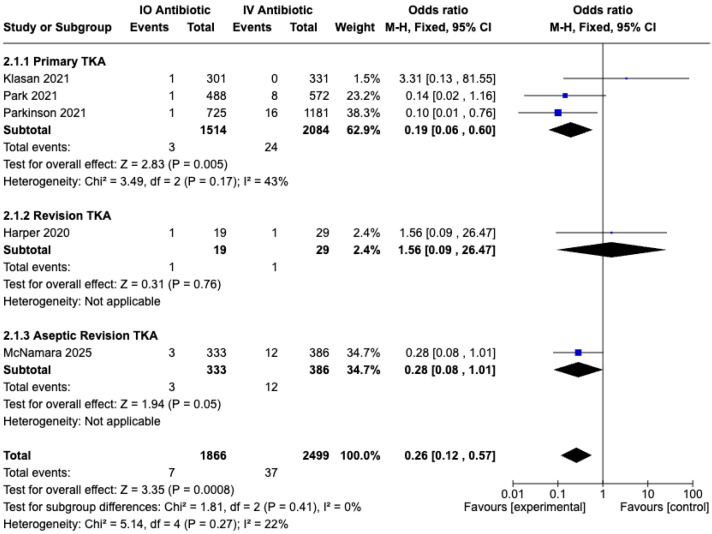
Forest plot comparing prosthesis joint infection (PJI) rate between IO and IV antibiotic prophylaxis groups [30,31,32,33,34].

A total of seven studies [30,31,32,33,34,35,36] reported the prosthesis joint infection (PJI) rate (Table 3). Five retrospective cohort studies [30,31,32,33,34], comprising 4516 cases, were included in the meta-analysis (Table 1 and Table 3). Pooled data from these studies demonstrated a significantly lower PJI rate in the IO group compared with the IV group (OR, 0.26; 95% CI, 0.12–0.57; *p* = 0.0008; I^2^ = 46%; fixed effects model) (Figure 5).

As a subgroup analysis, we separately evaluated the revision TKA group and the aseptic revision TKA group apart from the primary TKA group. In the primary TKA subgroup, a significant reduction in PJI was observed (OR, 0.19; 95% CI, 0.06 to 0.60; *p* = 0.005; I^2^ = 43%; fixed effects model) (Figure 5). In contrast, the revision TKA subgroup did not demonstrate a significant effect (*p* = 0.76). However, the aseptic revision subgroup showed a borderline statistically significant effect (*p* = 0.05).

In addition to the meta-analysis, Lachiewicz et al. [35]. reported no protective effect of IO vancomycin in 20 aseptic revision TKAs, In this prospective single-surgeon series, IO vancomycin infusion did not confer a protective effect against early prosthetic joint infection, with three cases of infection (15%) occurring within a mean follow-up of 10 months. However, Christopher et al. [36] observed a markedly lower PJI rate (0.88% at the final follow-up of ≥1 year) in 117 aseptic revision TKAs with IO vancomycin, suggesting a potential benefit in larger cohorts.

#### 3.5.2. Prosthesis Joint Infection (PJI) Stratified by Causative Pathogens

**Figure 6:**
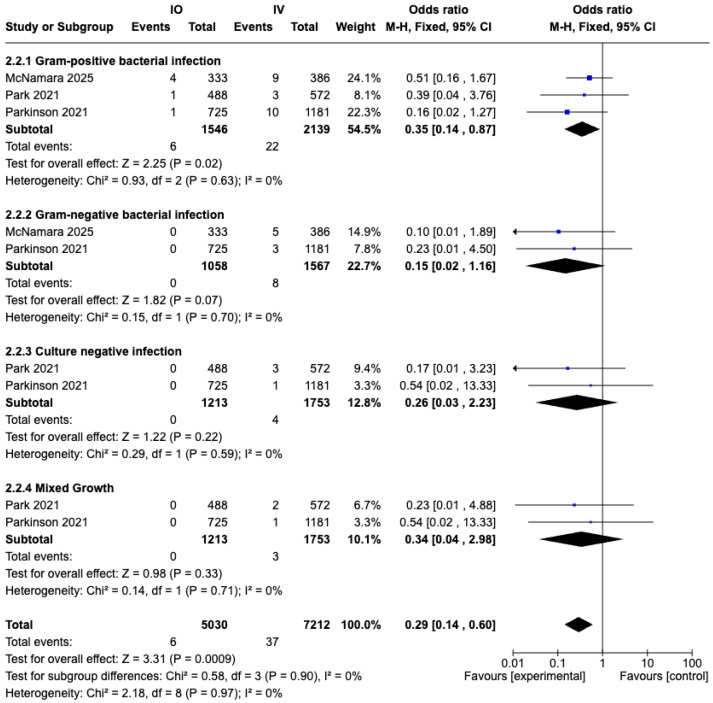
Forest plot comparing prosthesis joint infection (PJI) rate stratified by causative pathogens between IO and IV antibiotic prophylaxis groups [31,33,34].

A total of four studies [31,33,34,35] reported data on causative pathogens of prosthetic joint infection (PJI) (Table 3). Three retrospective cohort studies [31,33,34], comprising 2966 cases, were included in the meta-analysis (Table 1 and Table 3). The analysis was stratified into four subgroups (Gram-positive bacterial infection, Gram-negative bacterial infection, culture-negative infection, and mixed growth) to examine the association between vancomycin use and causative pathogens. Among these, a significant reduction in PJI was observed in the IO group compared with the IV group for Gram-positive bacterial infections (OR, 0.35; 95% CI, 0.14 to 0.87; *p* = 0.02; I^2^ = 0%; fixed effects model) (Figure 6).

In the study by Lachiewicz et al. [35], intraosseous vancomycin infusion conferred no protective benefit, with three early PJIs occurring—two caused by Gram-negative organisms and one by coagulase-negative Staphylococcus. Although this small cohort did not demonstrate overall efficacy, the absence of Methicillin-resistant Staphylococcus aureus infections is consistent with the findings of our meta-analysis.

#### 3.5.3. Complications

**Figure 7:**
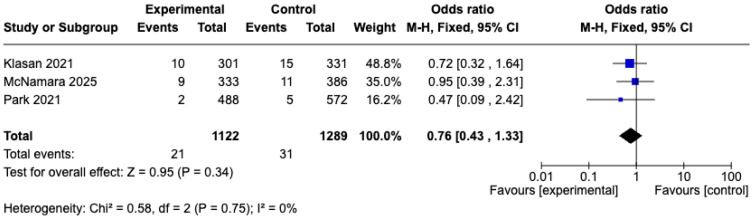
Forest plot comparing the incidence rate of acute kidney injury (AKI) between IO and IV antibiotic prophylaxis groups [32,33,34].

We extracted overall complication-related outcomes, including acute kidney injury (AKI), pulmonary embolism (PE), deep vein thrombosis (DVT), and red man syndrome (RMS), but only AKI could be subjected to meta-analysis (Table 3). A total of three studies [32,33,34] reported data on the incidence of AKI, of which three retrospective cohort studies [19,21,22], comprising 2411 cases, were included in the analysis (Table 1, Table 2 and Table 3). The pooled results demonstrated no significant difference between the IO and IV groups (OR, 0.76; 95% CI, 0.43 to 1.33; *p* = 0.34; I^2^ = 0%; fixed effects model) (Figure 7).

## 4. Discussion

The use of vancomycin for intraosseous administration was first introduced by Young et al. [23]. Prior to the adoption of vancomycin, cephalosporins were widely used in TKA procedures, and they substantially reduced the incidence of deep infections [6,37,38]. However, the growing problem of antibiotic resistance has necessitated the search for alternative prophylactic agents, with vancomycin emerging as a potential option [7,8,39]. Vancomycin has gained attention for its sensitivity against Methicillin-resistant Staphylococcus aureus (MRSA) and coagulase-negative staphylococci (CoNS) in achieving adequate MIC levels [9]. Nevertheless, vancomycin has also been associated with adverse effects, including infusion rate-related red man syndrome (RMS) and an increased risk of renal and other systemic toxicities, including acute kidney injury (AKI) [40]. In this context, intraosseous administration of vancomycin has been suggested to reduce systemic concentrations and thereby minimize complications, while achieving high local antibiotic levels that may help prevent prosthetic joint infection [23].

This systematic review aimed to examine the effectiveness and safety of intraosseous (IO) regional antibiotic prophylaxis through the assessment of tissue and serum concentrations, PJI rate, and complication incidence. In the meta-analysis, we synthesized data from 15 studies—including 8 randomized controlled trials (RCTs), 6 retrospective cohort studies, and 1 prospective cohort study— encompassing 4873 TJA cases. In the majority of the reviewed studies, vancomycin was the antibiotic of choice for IO administration.

The results of this meta-analysis confirm that IO administration achieved significantly higher local tissue concentrations and lower serum concentrations compared with IV administration, even without the use of a tourniquet in Wininger et al. [29]. Although lower serum concentrations were expected to reduce the risk of systemic toxicity, particularly the incidence of acute kidney injury, this was not statistically demonstrated when compared with IV administration. The results also demonstrated a reduction in PJI incidence, with a similar trend confirmed in the subgroup analysis of aseptic revision TKA. Pathogen-stratified meta-analysis revealed that a significant reduction in PJI rates was observed exclusively in cases caused by Gram-positive bacteria. In addition, the most recent study by McNamara et al. [34] demonstrated a reduction in PJI rates (*p* = 0.04), supporting the significant sensitivity of vancomycin against Gram-positive organisms, including MRSA. Data regarding tourniquet-related complications, including pulmonary embolism (PE) and deep vein thrombosis (DVT), were extracted; however, most studies either reported no such events or did not report them at all (Table 2 and Table 3). Nevertheless, in the recent study by McNamara et al. [34], which included 719 cases, the IO group demonstrated lower complication rates compared with the IV group (DVT: 1.2% vs. 1.8%; PE: 0% vs. 0.3%).

The most recent systematic review by Viswanathan et al. [12], which included 11 studies (1 prospective series, 6 RCTs, and 4 retrospective studies), reported that IO access provided substantially enhanced antibiotic penetration into local tissues, with significantly higher concentrations in bone (*p* = 0.0009) and fat (*p* = 0.03). In meta-analyses evaluating PJI mitigation, a significant reduction in PJI rates was observed in primary TKA; however, when revision TKA cases were included, the overall effect did not reach statistical significance (*p* = 0.23). The incidence of isolated Gram-positive infections was significantly higher in the IV group compared with the IO prophylaxis group. In another systematic review, Yu et al. [13] included 4091 patients across 12 studies (7 RCTs, 4 retrospective cohort studies, and 1 prospective cohort study). The authors reported that intraosseous infusion of 500 mg of vancomycin significantly increased antibiotic concentrations in periarticular adipose tissue (*p* < 0.001) and bone tissue (*p* < 0.001) compared with 1 g administered intravenously. A meta-analysis of postoperative PJI incidence was conducted exclusively in primary TKA, demonstrating that IO administration of 500 mg of vancomycin was more effective in reducing PJI rates compared with 1 g administered intravenously (*p* < 0.001). Additionally, no significant differences were observed between the two groups regarding postoperative pulmonary embolism (*p* = 0.59) or vancomycin-related complications (*p* = 0.44).

In comparison with previous reviews, this study demonstrates several notable strengths. It constitutes the largest and most comprehensive meta-analysis on intraosseous antibiotic prophylaxis in TJA to date. Compared with the earlier review by Viswanathan et al. [12], this analysis included two additional randomized controlled trials [28,29] and two retrospective studies [30,34]. The inclusion of these newly added studies enabled, for the first time, a quantitative meta-analysis of outcomes such as serum antibiotic concentrations and aseptic revision TKA, which had not been previously evaluated. This study also adhered strictly to the PRISMA guidelines and the Cochrane Handbook, with risk of bias assessed using the ROB-2 tool for randomized controlled trials and the ROBINS-I for non-randomized studies, thereby ensuring a systematic and rigorous appraisal of the evidence base.

This technique allows for the delivery of antibiotic concentrations well above the MIC directly at the surgical site, avoiding the logistical issues associated with timing IV infusions, which is a known challenge with agents like vancomycin. The approach is well-suited for fast-track protocols and could be especially valuable for high-risk patients, such as those with MRSA colonization, obesity, or diabetes [41]. From a health–economic standpoint, the observed reduction in PJI rates could justify the widespread adoption of IO prophylaxis, provided that costs are managed. For successful implementation, standardized protocols, staff training, and careful postoperative monitoring of renal function in at-risk patients are essential.

Future research should be directed at addressing these gaps. There is a need for more comparative trials, particularly head-to-head comparisons against dual IV antibiotic prophylaxis. Further studies are required to establish optimal dosing strategies and to confirm the safety and efficacy of IO prophylaxis in revision and hip arthroplasty. Long-term follow-up extending beyond one year is crucial to evaluate the sustained effect on infection prevention and to identify any potential late adverse events. Finally, research into the cost-effectiveness of this technique within enhanced recovery programs and translational studies on novel IO delivery formulations would help to further optimize prophylactic strategies in total joint arthroplasty.

## 5. Conclusions

Intraosseous (IO) antibiotic prophylaxis demonstrates significantly higher local antibiotic concentrations in bone and soft tissues and effectively reduces prosthetic joint infection (PJI) rates compared with intravenous administration. This benefit was consistently observed across primary and aseptic revision TKA. No significant increase in systemic complications was reported, supporting the safety and clinical utility of IO prophylaxis in both primary and revision arthroplasty settings.

## Figures and Tables

**Table 1:** Main characteristics of the included studies.

Study	Year	Design	IO						IV					
			Number	Mean Age	Female (%)	Average BMI	Tourniquet Time (min)	Minimum Follow-Up (Months)	Number	Mean Age	Female (%)	Average BMI	Tourniquet Time (min)	Minimum Follow-Up (Months)
Young et al. [11]	2013	RCT	11	71.8	45.5%	27.7	84	12	11	65.3	63.6%	29.1	82	12
(A) Young et al. [23]	2014	RCT	10	70.8	50%	32.2	105	-	10	71.4	90%	34.8	99	-
(B) Young et al. [23]	2014	RCT	10	71.7	60%	30	102	-						
Chin et al. [24]	2018	RCT	11	66	36.4%	41.1	For entire procedure	6	11	63	45.5%	40.1	For entire procedure	6
Young et al. [25]	2018	RCT	10	68	50%	33	129	-	10	69	70%	32	126	-
(A) Harper et al. [30]	2020	Cohort ^R^	100	67	53%	32	+1.87 min than IV	3	100	67	60%	32	-	3
(B) Harper et al. [30]	2020	Cohort ^R^	19	66	53%	32	-	3	29	69	59%	32	-	3
Parkinson et al. [31]	2021	Cohort ^R^	725	67	52%	31.5	At least 30 min	12	1181	67	49%	31.3	At least 30 min	12
Klasan et al. [32]	2021	Cohort ^R^	301	67.7	58.5%	31.8	-	12	331	68.7	57.1%	31.4	-	12
Park et al. [33]	2021	Cohort ^R^	448	67.4	58.6%	32.0	+2 min than IV	3	572	66.7	57.7%	32.5	-	3
Spangehl et al. [26]	2022	RCT	12	69	41.7%	32	25	-	12	68	58.3%	33	15	-
Harper et al. [27]	2023	RCT	10	67.9	30%	27.9	-	3	10	67.3	20%	27.4	-	3
Lachiewicz et al. [35]	2023	Cohort ^P^	20	67	10%	34.4	-	3	-	-	-	-	-	-
Zhang et al. [28]	2024	RCT	30	68.9	80%	25,9	75	-	30	67.8	76.7%	25.6	-	-
Wininger et al. [29]	2024	RCT	10	69	70%	29.4	0	3	10	67	50%	31	0	3
Christopher et al. [36]	2024	Cohort ^R^	117	68	53%	30.2	-	12	-	-	-	-	-	-
McNamara et al. [34]	2025	Cohort ^R^	333	67	66.4%	32.7	66.3	3	386	66	58.8	33.2	66.2	3

^R^ = retrospective study; ^P^ = prospective study

**Table 2:** Outcomes related to antibiotic concentrations and complication in included studies.

Study	Year	Operation	IO					IV				
			Dosage	Bone (μg/g)	Fat (μg/g)	Serum (μg/mL)	Complication	Dosage	Bone (μg/g)	Fat (μg/g)	Serum (μg/mL)	Complication
Young et al. [11]	2013	TKA	1 g Cefazolin	130	186	-	No	1 g Cefazolin	11.4	10.6	-	No
(A) Young et al. [23]	2014	TKA	250 mg Vancomycin	16	14	-	No	1 g Vancomycin	4.0	3.2	-	No
(B) Young et al. [23]	2014	TKA	500 mg Vancomycin	38	44	-	1 DVT					
Chin et al. [24]	2018	TKA	500 mg Vancomycin	34.3	39.3	1.8	1 PE	15 mg/kg Vancomycin	6.1	4.4	16.6	No
Young et al. [25]	2018	Aseptic rTKA	500 mg Vancomycin	63	18	-	No	1 g Vancomycin	7.1	3.6	-	No
Spangehl et al. [26]	2022	TKA	500 mg Vancomycin	25.3	40.3	4.9	No	15 mg/kg Vancomycin	8.6	6	18.9	No
Harper et al. [27]	2023	THA	500 mg Vancomycin	130.9	-	5.8	No	15 mg/kg Vancomycin	68	-	21	No
Zhang et al. [28]	2024	TKA	2 g Cefazolin	-	247.9	-	No	2 g Cefazolin	-	11.4	-	No
Wininger et al. [29]	2024	TKA	500 mg Vancomycin	66.2	-	7.8	0	15 mg/kg Vancomycin	61	-	19.6	0

**Table 3:** Outcomes related to PJI rate and complication in included studies.

Study	Year	Operation	IO			IV		
			Dosage	PJI Rate (%)	Complication	Dosage	PJI Rate (%)	Complication
(A) Harper et al. [30]	2020	TKA	500 mg Vancomycin	0	0	15 mg/kg Vancomycin	0	1 DVT
(B) Harper et al. [30]	2020	rTKA	500 mg Vancomycin	5.3	No	15 mg/kg Vancomycin	3.4	No
Parkinson et al. [31]	2021	TKA	1 g Cefazolin(46%) or 500 mg Vancomycin (54%)	0.1	No	2 g Cefazolin	1.4	No
Klasan et al. [32]	2021	TKA	500 mg Vancomycin	0.3	AKI: 3.0%	3 × 1 g Cefazolin	0	AKI: 5.0%
Park et al. [33]	2021	TKA	500 mg Vancomycin	0.22	No	15 mg/kg Vancomycin	1.5	No
Lachiewicz et al. [35]	2023	Aseptic rTKA	500 mg Vancomycin	15%	No	-	-	-
Christopher et al. [36]	2024	Aseptic rTKA	500 mg Vancomycin	0.88%	No	-	-	-
McNamara et al. [34]	2025	Aseptic rTKA	500 mg Vancomycin	0.9	DVT: 1.2% PE: 0% AKI: 2.7%	15 mg/kg Vancomycin	3.1	DVT: 1.8% PE: 0.3% AKI: 2.9%

## Data Availability

All data from this study are available on reasonable request to the corresponding author.

## Supplementary Materials

The following supporting information can be downloaded at https://www.mdpi.com/article/10.3390/medicina61101750/s1, Supplementary S1: Search strategy.

## Abbreviations

The following abbreviations are used in this manuscript:

THA	Total hip arthroplasty
TKA	Total knee arthroplasty
TJA	Total joint arthroplasty
RCT	Randomized controlled trial
IO	Intraosseous
IV	Intravenous
BMI	Body mass index
PJI	Prosthesis joint infection
AKI	Acute kidney injury
PE	Pulmonary embolism
DVT	Deep vein thrombosis
RMS	Red man syndrome
