# Supplementary material for: Efficacy and Safety of Intraosseous Versus Intravenous Antibiotic in Primary and Revision Total Joint Arthroplasty: A Systematic Review and Meta-Analysis
Source: Medicina (Kaunas). 2025 Sep 25;61(10):1750. doi: 10.3390/medicina61101750 (PMC12565870; doi:10.3390/medicina61101750)
Supplement: Supplementary file 1 [file medicina-61-01750-s001.zip › medicina-3876232-supplementary.pdf]

Supplementary S1: Search strategy

Search strategy (Embase+PubMed)

Population #1

#1	'hip arthroplasty'/exp OR 'arthroplasty, hip' OR 'hip arthroplasties' OR 'hip arthroplasty' OR 'hip extracapsular arthroplasty' OR 'hip joint alloplasty' OR 'hip joint arthroplasty' OR 'hip plasty' OR 'hiparthroplasty' OR 'knee arthroplasty'/exp OR 'arthroplasty, knee' OR 'knee arthroplasties' OR 'knee arthroplasty' OR 'knee reconstruction' OR 'reconstruction, knee' OR 'hip replacement'/exp OR 'arthroplasty, replacement, hip' OR 'hip joint replacement' OR 'hip joint replacements' OR 'hip replacement' OR 'hip replacement arthroplasty' OR 'hip replacements' OR 'revision arthroplasty'/exp OR 'arthroplasty, revision' OR 'revision arthroplasty' OR 'arthroplasty'/exp OR 'alloarthroplasty' OR 'arthroplasties' OR 'arthroplasty' OR 'joint reconstruction' OR 'joint reconstructive procedure' OR 'joint reconstructive surgery'
#2	('antibiotic agent'/exp OR 'antibiotic' OR 'antibiotic agent' OR 'antibiotic combination' OR 'antibiotic drug' OR 'antibiotic ointment' OR 'antibiotic residue' OR 'antibiotic spectrum' OR 'antibiotics' OR 'antibiotics and their derivatives' OR 'antibiotics, combined' OR 'antibiotics, folate antagonists' OR 'antibiotics, miscellaneous' OR 'antibiotics, nitrofuran' OR 'antibiotics, oxalodinones' OR 'combined antibiotic') OR ('topical antiinfective agent'/exp OR 'anti infective agents, local' OR 'anti infectives, topical' OR 'anti-infective agents, local' OR 'anti-infectives, topical' OR 'antiinfectives, topical' OR 'local anti infective agent' OR 'local antiinfective agent' OR 'mercurial antiseptic, organic' OR 'organic mercurial antiseptic' OR 'topical antiinfective' OR 'topical antiinfective agent' OR 'topical antiinfectives') OR ('antibiotic prophylaxis'/exp OR 'antibiotic prevention' OR 'antibiotic prophylaxis' OR 'prevention, antibiotic' OR 'prophylaxis, antibiotic') OR ('antibiotic therapy'/exp OR 'anti-bacterial therapy' OR 'anti-bacterial treatment' OR 'anti-biotic therapy' OR 'anti-biotic treatment' OR 'antibacterial therapy' OR 'antibacterial treatment' OR 'antibiotic chemotherapy' OR 'antibiotic therapy' OR 'antibiotic treatment' OR 'therapy, antibiotic')

#3	'intraosseous drug administration'/exp OR 'IO administration' OR 'IO drug administration' OR 'IO infusion' OR 'IO injection' OR 'bone infusion' OR 'bone injection' OR 'drug administration, bone' OR 'drug administration, intraosseous' OR 'infusions, intraosseous' OR 'intra-bone drug administration' OR 'intra-bone marrow injection' OR 'intra-osseous administration' OR 'intra-osseous infusion' OR 'intra-osseous injection' OR 'intrabone injection' OR 'intrabone marrow injection' OR 'intraosseous administration' OR 'intraosseous application' OR 'intraosseous drug administration' OR 'intraosseous infusion' OR 'intraosseous infusions' OR 'intraosseous injection'
#4	#1 AND #2 AND #3

Search strategy (Scopus)

#1	(TITLE-ABS-KEY("hip arthroplasty") OR TITLE-ABS-KEY("arthroplasty, hip") OR TITLE-ABS-KEY("hip arthroplasties") OR TITLE-ABS-KEY("hip extracapsular arthroplasty") OR TITLE-ABS-KEY("hip joint alloplasty") OR TITLE-ABS-KEY("hip joint arthroplasty") OR TITLE-ABS-KEY("hip plasty") OR TITLE-ABS-KEY("hiparthroplasty") OR TITLE-ABS-KEY("knee arthroplasty") OR TITLE-ABS-KEY("arthroplasty, knee") OR TITLE-ABS-KEY("knee arthroplasties") OR TITLE-ABS-KEY("knee arthroplasty") OR TITLE-ABS-KEY("knee reconstruction") OR TITLE-ABS-KEY("reconstruction, knee") OR TITLE-ABS-KEY("hip replacement") OR TITLE-ABS-KEY("arthroplasty, replacement, hip") OR TITLE-ABS-KEY("hip joint replacement") OR TITLE-ABS-KEY("hip joint replacements") OR TITLE-ABS-KEY("hip replacement arthroplasty") OR TITLE-ABS-KEY("hip replacements") OR TITLE-ABS-KEY("revision arthroplasty") OR TITLE-ABS-KEY("arthroplasty, revision") OR TITLE-ABS-KEY("arthroplasty") OR TITLE-ABS-KEY("alloarthroplasty") OR TITLE-ABS-KEY("arthroplasties") OR TITLE-ABS-KEY("joint reconstruction") OR TITLE-ABS-KEY("joint reconstructive procedure") OR TITLE-ABS-KEY("joint reconstructive surgery"))

#2	(TITLE-ABS-KEY("antibiotic") OR TITLE-ABS-KEY("antibiotic agent") OR TITLE-ABS-KEY("antibiotic combination") OR TITLE-ABS-KEY("antibiotic drug") OR TITLE-ABS-KEY("antibiotic ointment") OR TITLE-ABS-KEY("antibiotic residue") OR TITLE-ABS-KEY("antibiotic spectrum") OR TITLE-ABS-KEY("antibiotics") OR TITLE-ABS-KEY("antibiotics and their derivatives") OR TITLE-ABS-KEY("antibiotics, combined") OR TITLE-ABS-KEY("antibiotics, folate antagonists") OR TITLE-ABS-KEY("antibiotics, miscellaneous") OR TITLE-ABS-KEY("antibiotics, nitrofuran") OR TITLE-ABS-KEY("antibiotics, oxalodinones") OR TITLE-ABS-KEY("combined antibiotic") OR TITLE-ABS-KEY("anti infective agents, local") OR TITLE-ABS-KEY("anti infectives, topical") OR TITLE-ABS-KEY("anti-infective agents, local") OR TITLE-ABS-KEY("anti-infectives, topical") OR TITLE-ABS-KEY("antiinfectives, topical") OR TITLE-ABS-KEY("local anti infective agent") OR TITLE-ABS-KEY("local antiinfective agent") OR TITLE-ABS-KEY("mercurial antiseptic, organic") OR TITLE-ABS-KEY("organic mercurial antiseptic") OR TITLE-ABS-KEY("topical antiinfective") OR TITLE-ABS-KEY("topical antiinfective agent") OR TITLE-ABS-KEY("topical antiinfectives") OR TITLE-ABS-KEY("antibiotic prevention") OR TITLE-ABS-KEY("antibiotic prophylaxis") OR TITLE-ABS-KEY("prevention, antibiotic") OR TITLE-ABS-KEY("prophylaxis, antibiotic") OR TITLE-ABS-KEY("anti-bacterial therapy") OR TITLE-ABS-KEY("anti-bacterial treatment") OR TITLE-ABS-KEY("anti-biotic therapy") OR TITLE-ABS-KEY("anti-biotic treatment") OR TITLE-ABS-KEY("antibacterial therapy") OR TITLE-ABS-KEY("antibacterial treatment") OR TITLE-ABS-KEY("antibiotic chemotherapy") OR TITLE-ABS-KEY("antibiotic therapy") OR TITLE-ABS-KEY("antibiotic treatment") OR TITLE-ABS-KEY("therapy, antibiotic"))
#3	(TITLE-ABS-KEY("IO administration") OR TITLE-ABS-KEY("IO drug administration") OR TITLE-ABS-KEY("IO infusion") OR TITLE-ABS-KEY("IO injection") OR TITLE-ABS-KEY("bone infusion") OR TITLE-ABS-KEY("bone injection") OR TITLE-ABS-KEY("drug administration, bone") OR TITLE-ABS-KEY("drug administration, intraosseous") OR TITLE-ABS-KEY("infusions, intraosseous") OR TITLE-ABS-KEY("intra-bone drug administration") OR TITLE-ABS-KEY("intra-bone marrow injection") OR TITLE-ABS-KEY("intra-osseous administration") OR TITLE-ABS-KEY("intra-osseous infusion") OR TITLE-ABS-KEY("intra-osseous injection") OR TITLE-ABS-KEY("intrabone injection") OR TITLE-ABS-KEY("intrabone marrow injection") OR TITLE-ABS-KEY("intraosseous administration") OR TITLE-ABS-KEY("intraosseous application") OR TITLE-ABS-KEY("intraosseous drug administration") OR TITLE-ABS-KEY("intraosseous infusion") OR TITLE-ABS-KEY("intraosseous infusions") OR TITLE-ABS-KEY("intraosseous injection"))

#4	#1 AND #2 AND #3

Search strategy (Web of Science)

#1	TS=(hip arthroplasty OR arthroplasty, hip OR hip arthroplasties OR hip extracapsular arthroplasty OR hip joint alloplasty OR hip joint arthroplasty OR hip plasty OR hiparthroplasty OR knee arthroplasty OR arthroplasty, knee OR knee arthroplasties OR knee arthroplasty OR knee reconstruction OR reconstruction, knee OR hip replacement OR arthroplasty, replacement, hip OR hip joint replacement OR hip joint replacements OR hip replacement arthroplasty OR hip replacements OR revision arthroplasty OR arthroplasty, revision OR arthroplasty OR alloarthroplasty OR arthroplasties OR joint reconstruction OR joint reconstructive procedure OR joint reconstructive surgery)
#2	TS=(antibiotic OR antibiotic agent OR antibiotic combination OR antibiotic drug OR antibiotic ointment OR antibiotic residue OR antibiotic spectrum OR antibiotics OR antibiotics and their derivatives OR antibiotics, combined OR antibiotics, folate antagonists OR antibiotics, miscellaneous OR antibiotics, nitrofuran OR antibiotics, oxalodinones OR combined antibiotic OR anti infective agents, local OR anti infectives, topical OR anti-infective agents, local OR anti-infectives, topical OR antiinfectives, topical OR local anti infective agent OR local antiinfective agent OR mercurial antiseptic, organic OR organic mercurial antiseptic OR topical antiinfective OR topical antiinfective agent OR topical antiinfectives OR antibiotic prevention OR antibiotic prophylaxis OR prevention, antibiotic OR prophylaxis, antibiotic OR anti-bacterial therapy OR anti-bacterial treatment OR anti-biotic therapy OR anti-biotic treatment OR antibacterial therapy OR antibacterial treatment OR antibiotic chemotherapy OR antibiotic therapy OR antibiotic treatment OR therapy, antibiotic)

#3	TS=(IO administration OR IO drug administration OR IO infusion OR IO injection OR bone infusion OR bone injection OR drug administration, bone OR drug administration, intraosseous OR infusions, intraosseous OR intra-bone drug administration OR intra-bone marrow injection OR intra-osseous administration OR intra-osseous infusion OR intra-osseous injection OR intrabone injection OR intrabone marrow injection OR intraosseous administration OR intraosseous application OR intraosseous drug administration OR intraosseous infusion OR intraosseous infusions OR intraosseous injection)
#4	#1 AND #2 AND #3

Search strategy (Cochrane Library)

#1	MeSH descriptor: [Arthroplasty, Replacement, Hip] explode all trees
#2	(Arthroplasties, Hip Replacement OR Hip Replacement Arthroplasties OR Hip Prosthesis Implantations OR Hip Replacement Arthroplasty OR Arthroplasties, Replacement, Hip OR Arthroplasty, Hip Replacement OR Prosthesis Implantation, Hip OR Hip Prosthesis Implantation OR Replacement Arthroplasties, Hip OR Implantation, Hip Prosthesis OR Replacement Arthroplasty, Hip OR Arthroplasty, Total Hip OR Total Hip Replacements OR Total Hip Replacement OR Replacement, Total Hip OR Total Hip Arthroplasty OR Total Hip Arthroplasties OR Hip Replacement, Total OR Hip Arthroplasty, Total):ti,ab,kw
#3	MeSH descriptor: [Arthroplasty, Replacement, Knee] explode all trees

#4	(Unicompartmental Knee Arthroplasty OR Arthroplasty, Partial Knee OR Knee Replacement, Unicondylar OR Knee Arthroplasty, Unicompartmental OR Partial Knee Arthroplasty OR Knee Replacement, Unicompartmental OR Arthroplasty, Replacement, Partial Knee OR Unicondylar Knee Arthroplasty OR Arthroplasty, Unicompartmental Knee OR Knee Arthroplasty, Unicondylar OR Knee Arthroplasty, Partial OR Partial Knee Replacement OR Arthroplasty, Unicondylar Knee OR Unicompartmental Knee Replacement OR Knee Replacement, Partial OR Unicondylar Knee Replacement OR Replacement Arthroplasty, Knee OR Total Knee Replacement OR Arthroplasty, Knee OR Knee Arthroplasty OR Knee Arthroplasty, Total OR Arthroplasties, Replacement, Knee OR Arthroplasty, Total Knee OR Total Knee Arthroplasty OR Arthroplasties, Knee Replacement OR Knee Replacement Arthroplasty OR Arthroplasty, Knee Replacement OR Knee Replacement, Total OR Replacement, Total Knee OR Replacement Arthroplasties, Knee OR Knee Replacement Arthroplasties):ti,ab,kw
#5	#1 OR #2 OR #3 OR #4
#6	MeSH descriptor: [Anti-Bacterial Agents] explode all trees
#7	(Antibiotics OR Antibiotic OR Antibacterial Agent OR Agent, Anti-Bacterial OR Compound, Anti-Bacterial OR Compounds, Anti-Bacterial OR Anti-Bacterial Compounds OR Anti Bacterial Agent OR Anti Bacterial Compound OR Anti Bacterial Compounds OR Agent, Antibacterial OR Agents, Anti-Bacterial OR Antibacterial Agents OR Anti-Bacterial Compound OR Anti Bacterial Agents OR Anti-Bacterial Agent OR Agents, Antibacterial OR Bacteriocides OR Agents, Bacteriocidal OR Agent, Bacteriocidal OR Bacteriocidal Agents OR Bacteriocide OR Bacteriocidal Agent OR Agent, Antimycobacterial OR Agents, Antimycobacterial OR Antimycobacterial Agents OR Anti Mycobacterial Agent OR Agents, Anti-Mycobacterial OR Agent, Anti-Mycobacterial OR Anti-Mycobacterial Agents OR Anti Mycobacterial Agents OR Antimycobacterial Agent OR Anti-Mycobacterial Agent):ti,ab,kw
#8	MeSH descriptor: [Anti-Infective Agents, Local] explode all trees
#9	(Antiinfective Agents, Local OR Antiseptics OR Antiinfective Agents, Topical OR Topical Anti Infective Agents OR Local Anti Infective Agents OR Agents, Topical Anti-Infective OR Antiseptic OR Local Anti-Infective Agents OR Anti Infective Agents, Local OR Topical Anti-Infective Agents OR Topical Antiinfective Agents OR Agents, Local Anti-Infective OR Local Antiinfective Agents OR Anti Infective Agents, Topical OR Agents, Local Antiinfective OR Agents, Topical Antiinfective OR Anti-Infective Agents, Topical OR Topical Microbicides OR Microbicides, Topical OR Local Microbicides OR Microbicides, Local):ti,ab,kw

#10	MeSH descriptor: [Vancomycin] explode all trees
#11	(Vanco Azupharma OR Vancomicina Norman OR Vancomicina Chiesi OR Vancomicina Combino Phar OR Vancocin HCl OR Vancocine OR Vancomycin Lilly OR Vancocin OR Vancomycin Sulfate OR Sulfate, Vancomycin OR Vancomicina Abbott OR Vancomycin Phosphate, Decahydrate OR Vancomycin-ratiopharm OR Vancomycin Phosphate OR Vancomycine Dakota OR Vancomycin Hexal OR Vanco-saar OR Hydrochloride, Vancomycin OR Vancomycin Hydrochloride OR AB-Vancomycin OR VANCO-cell OR Diatracin):ti,ab,kw
#12	#6 OR #7 OR #8 OR #9 OR #10 OR #11
#13	MeSH descriptor: [Infusions, Intraosseous] explode all trees
#14	(Intraosseous Infusion OR Infusions, Intra Osseous OR Infusion, Intraosseous OR Intra-Osseous Infusions OR Infusion, Intra-Osseous OR Intra-Osseous Infusion OR Intraosseous Infusions OR Infusions, Intra-Osseous):ti,ab,kw
#15	#13 OR #14
#16	#5 AND #12 AND #15
